# Left atrial function assessed by speckle tracking echocardiography in anthracycline-induced cardiotoxicity: a case report

**DOI:** 10.1093/ehjcr/ytaa355

**Published:** 2020-11-12

**Authors:** Daisuke Sueta, Hiroki Usuku, Yui Kinoshita, Kenichi Tsujita

**Affiliations:** 1 Department of Cardiovascular Medicine, Graduate School of Medical Sciences, Kumamoto University, 1-1-1, Honjo, Chuo-ku, Kumamoto 860-8556, Japan; 2 Department of Molecular Laboratory, Graduate School of Medical Sciences, Kumamoto University, 1-1-1, Honjo, Chuo-ku, Kumamoto 860-8556, Japan

**Keywords:** CTRCD, Left atrial function, Echocardiography, Case report

## Abstract

**Background:**

The onset prevention and early diagnosis in cardiotoxicity due to cancer chemotherapy are important, and it is important to detect cardiac dysfunction at an early stage and start treatment to enhance the therapeutic effect.

**Case summary:**

A 31-year-old female with breast cancer received chemotherapy with epirubicin (400 mg/m^2^) and cyclophosphamide followed by docetaxel. Two months after the initiation of her chemotherapy, the left ventricular (LV) ejection fraction (LVEF) determined by echocardiography fell to 41.2%, and she was diagnosed with cancer therapy-related cardiac dysfunction (CTRCD). Three months after the initiation of cancer treatment, the peak velocity of late diastolic transmitral Doppler flow (A wave) became undetectable. Peak longitudinal strain (LS) and peak LS rate, which reflect left atrium (LA) reservoir function, gradually declined like the LVEF and LV-global LS (GLS). Seven months after the initiation of cancer treatment, she was diagnosed with acute decompensated heart failure. The changes in peak LS and peak LS at the onset were greater than those in LVEF and LV-GLS.

**Discussion:**

This is a case report suggesting that LA reservoir function might be a more sensitive indicator than LVEF or LV-GLS in detecting CTRCD and that LA booster function might be the earliest. Left atrium reservoir function might be a more sensitive than conventional LV pump function and optimal indicator in CTRCD.

## Introduction

When cardiotoxicity due to cancer chemotherapy becomes serious, its treatment is often difficult, cancer treatment itself is interrupted, and the cardiotoxicity may be prognostically significant. Therefore, onset prevention and early diagnosis are important, and regular monitoring is essential during cancer treatment. We have already reported a novel assessment of cancer therapy-related cardiac dysfunction (CTRCD) by cardiac computed tomography.^1^ The frequency of echocardiography^2^ and myocardial biomarker^3^ measurements when using anticancer drugs is recommended according to the risk of cancer treatment. Cardinale *et al*.^4^ reported that the earlier the start of treatment for cardiac dysfunction in patients with anthracycline-induced cardiomyopathy, the greater are the number of responders of cardiac function. Hence, in patients with cancer, it is important to detect cardiac dysfunction at an early stage and start treatment to enhance the therapeutic effect.

**Learning points**

- Left atrium (LA) reservoir function may be a more sensitive indicator than left ventricular ejection fraction or left ventricular-global longitudinal strain in detecting cancer therapy-related cardiac dysfunction (CTRCD) and that LA booster function may be the earliest.
- Left atrium reservoir function may be an optimal indicator in CTRCD.

Cardiac ultrasonography is the most common method for assessing cardiac function. Left ventricular (LV) ejection fraction (LVEF) is effective when functional myocardial damage becomes apparent, but it does not have sufficient sensitivity and specificity to detect myocardial damage caused by a potential antineoplastic agent at an early stage. It was shown that long-axis myocardial strain [global longitudinal strain (GLS)] by the two-dimensional speckle tracking (2DS) method can be an indicator of potential cardiac dysfunction. Attempts to detect myocardial damage earlier than LVEF by combining with the biomarker troponin have been made.^5^ Furthermore, in recent years, it has been possible to evaluate not only LV function but also left atrium (LA) function using the 2DS method^6^, and regarding its usefulness, it was revealed to be useful for predicting recurrence after catheter ablation for atrial fibrillation cases^7^ and for predicting cardiac events after acute myocardial infarction^8^, mitral regurgitation^9^, aortic valve stenosis,^10^ and heart failure with preserved ejection function.^11^^,^^12^ Although LA strain (LAS) findings have been already described in chemotherapy-treated patients,^13^^,^^14^ there are few reports that follow time courses of LAS findings. Herein, we report the measurements of LAS over time in a CTRCD case with anthracycline anticancer drugs.

## Timeline

**Timeline:**
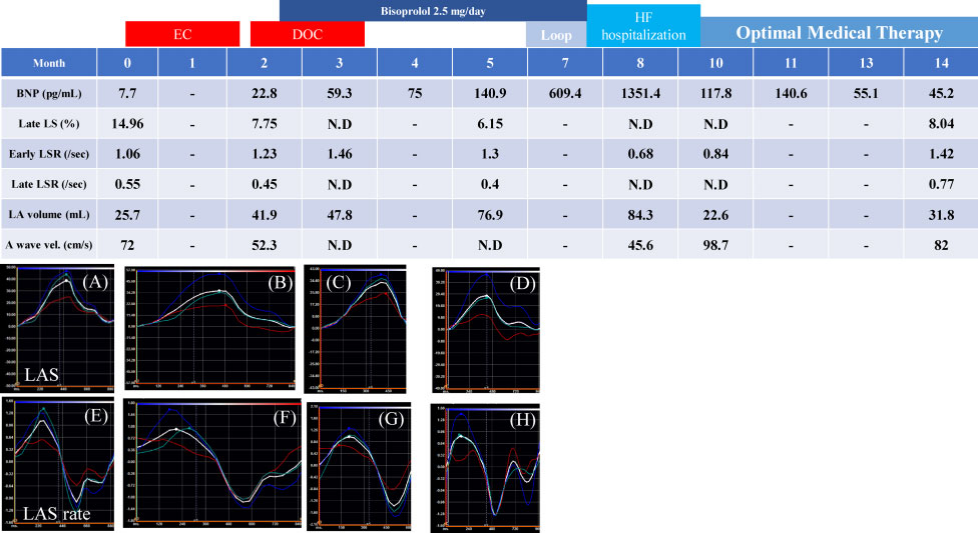
Clinical timeline describing the patient’s clinical presentation, including plasma brain natriuretic peptide levels and transthoracic echocardiography findings, and the treatment approach (upper panel). Lower figures demonstrate the left atrium waveform obtained by the two-dimensional speckle tracking method. (*A–D*) Left atrium strain and (*E–H*) left atrium strain rate. (*A* and *E*) Evaluation before cancer treatment, (*B* and *F*) 2 months after the initiation of cancer treatment, (*C* and *G*) 3 months after the initiation of cancer treatment, (*D* and *H*) 5 months after the initiation of cancer treatment. DOC, docetaxel administration; EC, epirubicin and cyclophosphamide administration; HF, heart failure; LA, left atrium; LAS, left atrium strain; Loop, loop diuretic (daily oral 10 mg furosemide administration); LS, longitudinal strain; LSR, longitudinal strain rate.

## Description of the case

A 31-year-old female patient received a diagnosis of poor-risk breast cancer. She had a smoking habit. She underwent total mastectomy and was indicated for postoperative chemotherapy. Pretreatment transthoracic echocardiography (TTE) revealed a low-normal LV function (LVEF, 50%). Adjuvant combination chemotherapy with epirubicin (total 400 mg/m^2^) and cyclophosphamide (total 2000 mg/m^2^) was administered every 3 weeks for four cycles. Then, she was administered docetaxel (total 300 mg/m^2^) every 3 weeks for four cycles.

Echocardiography was performed using commercially available ultrasound equipment. Left ventricular ejection fraction was assessed with the modified Simpson’s method using apical two- and four-chamber views. Peak velocities of early (E) and late (A) diastolic transmitral Doppler flow were measured using the apical four-chamber view with the sample volume placed at the tip of the mitral leaflets. We obtained echocardiographic images several ultrasound vendors (frame rate 43–50 Hz) and performed the 2DS tracking echocardiography analysis using TomTec Image-Arena™ (vendor-independent, TomTec Imaging Systems, German) software. Speckles were tracked frame by frame throughout the LV myocardium over the course of one cardiac cycle; basal, mid, and apical regions of interest were then created and were manually adjusted whenever needed. Because dedicated software for LAS analysis has not yet been released, we also used this software to study LA deformation. Left atrium strain was measured using strain indices calculated as the average of the three segments (left wall, roof, and right wall) obtained using apical four-chamber views. We used R-wave trigger method. All sonographers were blinded to the patient’s clinical history and data to minimize bias.

**Figure 1:**
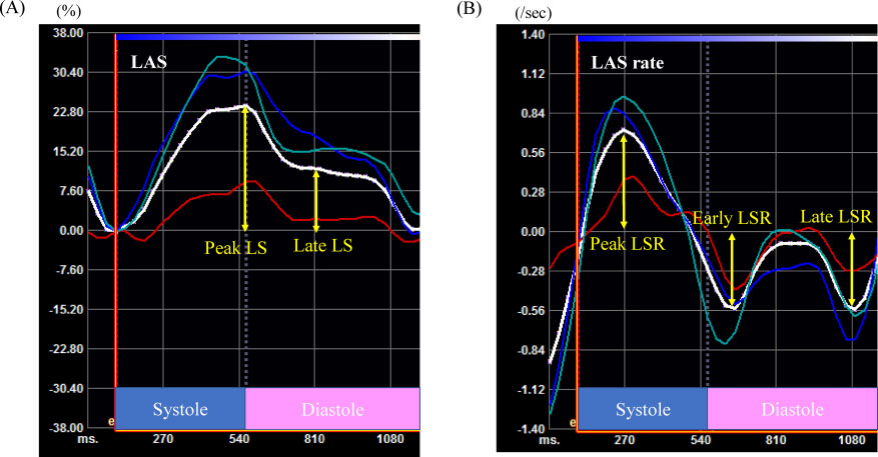
Typical measurement examples of the left atrium strain (*A*) and left atrium strain rate (*B*) evaluated by the two-dimensional speckle tracking method. Green, red, blue, and white curves indicate the left atrium free wall side, roof, septal wall side, and their average, respectively. LAS, left atrium strain; LS, longitudinal strain; LSR, longitudinal strain rate.

*Figure 1* demonstrates typical measurement examples of the LAS (*Figure 1A*) and LAS rate (*Figure 1B*) evaluated by the 2DS method. The systolic strain [peak longitudinal strain (LS) in *Figure 1A* and peak LS rate (LSR) in *Figure 1B*] reflects passive fullness and active relaxation, that is, LA reservoir function. The end-diastolic strain (late LS in *Figure 1A* and late LSR in *Figure 1B*) reflects atrial booster function. Early LSR reflects LA conduit function. These concepts are comprehensively reviewed.^15^

**Figure 2:**
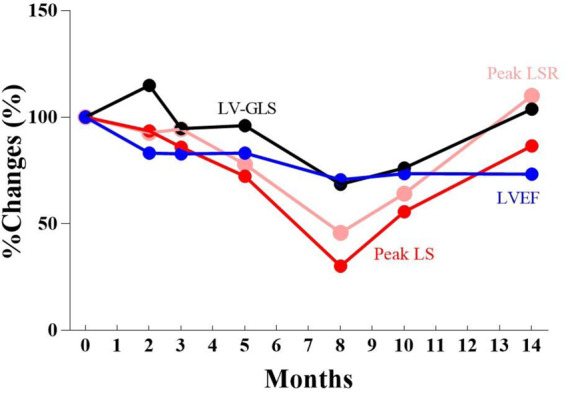
Serial percent changes in the transthoracic echocardiography findings [left ventricular ejection fraction (blue line), left ventricular-global longitudinal strain (black line), peak longitudinal strain (red line), and peak longitudinal strain rate (pink line)]. LS, longitudinal strain; LSR, longitudinal strain rate; LV-GLS, left ventricular-global longitudinal strain; LVEF, left ventricular ejection fraction.

As shown in *Figure 2*, 2 months after the initiation of her chemotherapy, LVEF determined by TTE fell to 41.2%, and she was diagnosed with CTRCD according to diagnostic criteria based on expert consensus from the American Society of Echocardiography and the European Association of Cardiovascular Imaging.^16^

Three months after the initiation of her chemotherapy, the patient remained asymptomatic, but her plasma brain natriuretic peptide (BNP) level was elevated (7.7 pg/mL→59.3 pg/mL) (normal range: <18.4 pg/mL). Thus, oral 2.5 mg bisoprolol fumarate daily administration was initiated. Seven months after the initiation of her chemotherapy, her plasma BNP level further increased, and an oral loop diuretic, furosemide 10 mg daily, was additionally administered. Peak LS and peak LSR, which are indicators of LA reservoir function, gradually declined like LVEF and LV-GLS. Eight months after the initiation of her chemotherapy, she was admitted to a hospital and diagnosed with acute decompensated heart failure (ADHF). Her body height and weight were 165.5 cm and 68.8 kg, respectively. Her blood pressure was 118/74 mm Hg, pulse 84/min, respiratory rate of 12/min with an O_2_ saturation of 98% on room air. On physical examination, there was no evidence of oedema and jugular venous distention. Her cardiovascular examination was normal, lungs were clear to auscultation. The percent change in peak LS and peak LSR at the time of ADHF onset was greater than LVEF and LV-GLS. Multidisciplinary treatment was performed, and optimal medical therapy, including a renin-angiotensin system inhibitor, was initiated.

As shown in the upper panel in timeline, 3 months after the initiation of cancer treatment, late LS and late LSR, which are indicators of atrial booster function, became undetectable [(B) and (F)] and the A wave became undetectable. This means that the LA booster function has fallen into a ‘loss-of-function’. The lower panels in timeline demonstrate the actual waveform obtained by TTE.

No further additional heart failure had occurred at regular medical check-ups.

## Discussion

In this case, as shown in *Figure 2*, it was suggested that the LA reservoir function might be a more sensitive indicator than LVEF and LV-GLS in detecting CTRCD. Furthermore, as shown in timeline, LA booster function has been lost, and this appears only 3 months after the initiation of anticancer treatment, which might be the earliest indicator. What is most striking is the early increase in LA volume, which reflects LA emptying fraction, noted only 2 months after commencement of chemotherapy and continuing to increase until the onset of ADHF.

Peak LS and peak LSR have been reported to correlate well with LV end-diastolic pressure (LVEDP),^17^ suggesting that they are useful indicators for predicting cardiac events. Cameli *et al*.^18^ examined the LA tissue of mitral regurgitation cases and found that peak LS and LA fibrosis were significantly correlated. Therefore, peak LS can be said to be an index that can non-invasively estimate LVEDP and LA fibrosis. We expected that a lower LVEF would increase intra-LV pressure and that LA booster function would therefore also decline. In fact, LA booster function was lost first, and then LA reservoir function decreased at the same time as LVEF and LV-GLS decreased. In the present case, peak LS also decreased, and it can be inferred that LVEDP decreased and LA reservoir function decreased. Thus, we believe that the phenomenon observed in the present case may be direct damage to the LA by anticancer agents. In other words, it is highly possible that LA damage caused by anticancer agents resulted in the decline of LA function. Cardinale *et al*.^19^ reported that the median time elapsed between the end of chemotherapy and cardiotoxicity development was 3.5 months, this case is consistent with their results, but the LA might be affected earlier. Hence, further pathophysiological and molecular physiological studies, including animal experiments, are warranted. Additional detailed, prospective, large-scale, long-term surveillance may be required to verify our theories.

The only limitation of this study was that we were unable to observe intracardiac pressure using cardiac catheterization. It is not realistic to perform frequent catheterizations during anticancer treatment. Therefore, this phenomenon was observed using TTE, which may have been reasonable.

This is a case report suggesting that LA reservoir function might be a more sensitive indicator than LVEF or LV-GLS in detecting CTRCD and that LA booster function might be the earliest, although LA pump strain value should be read in light of underlying LA volume.^20^ A wave velocity might be a second indicator of CTRCD.

In conclusion, LA reservoir function might be a more sensitive than conventional LV pump function and optimal indicator in CTRCD.

## Lead author biography

**Figure:**
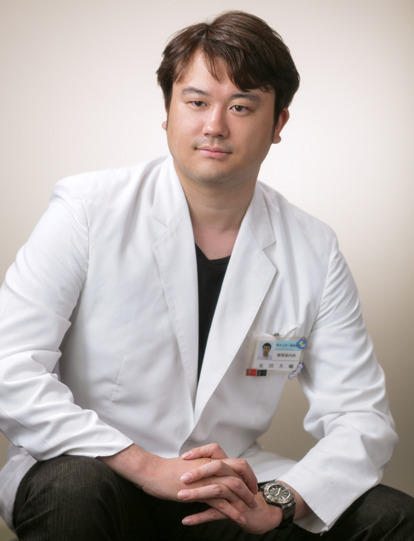


Daisuke Sueta is a Lecturer, Department of Cardiovascular Medicine, Kumamoto University Hospital.

## Supplementary material

Supplementary material is available at *European Heart Journal - Case Reports* online.

## Supplementary Material

ytaa355_Supplementary_Data
