# Supplementary material for: Left atrial function assessed by speckle tracking echocardiography in anthracycline-induced cardiotoxicity: a case report
Source: Eur Heart J Case Rep. 2020 Nov 12;4(6):1–5. doi: 10.1093/ehjcr/ytaa355 (PMC7793052; doi:10.1093/ehjcr/ytaa355)
Supplement: ytaa355_Supplementary_Data [file ytaa355_supplementary_data.zip › ytaa355-suppl_data/EHJ-CR-Slide-Set revised.pptx]

## Slide 1
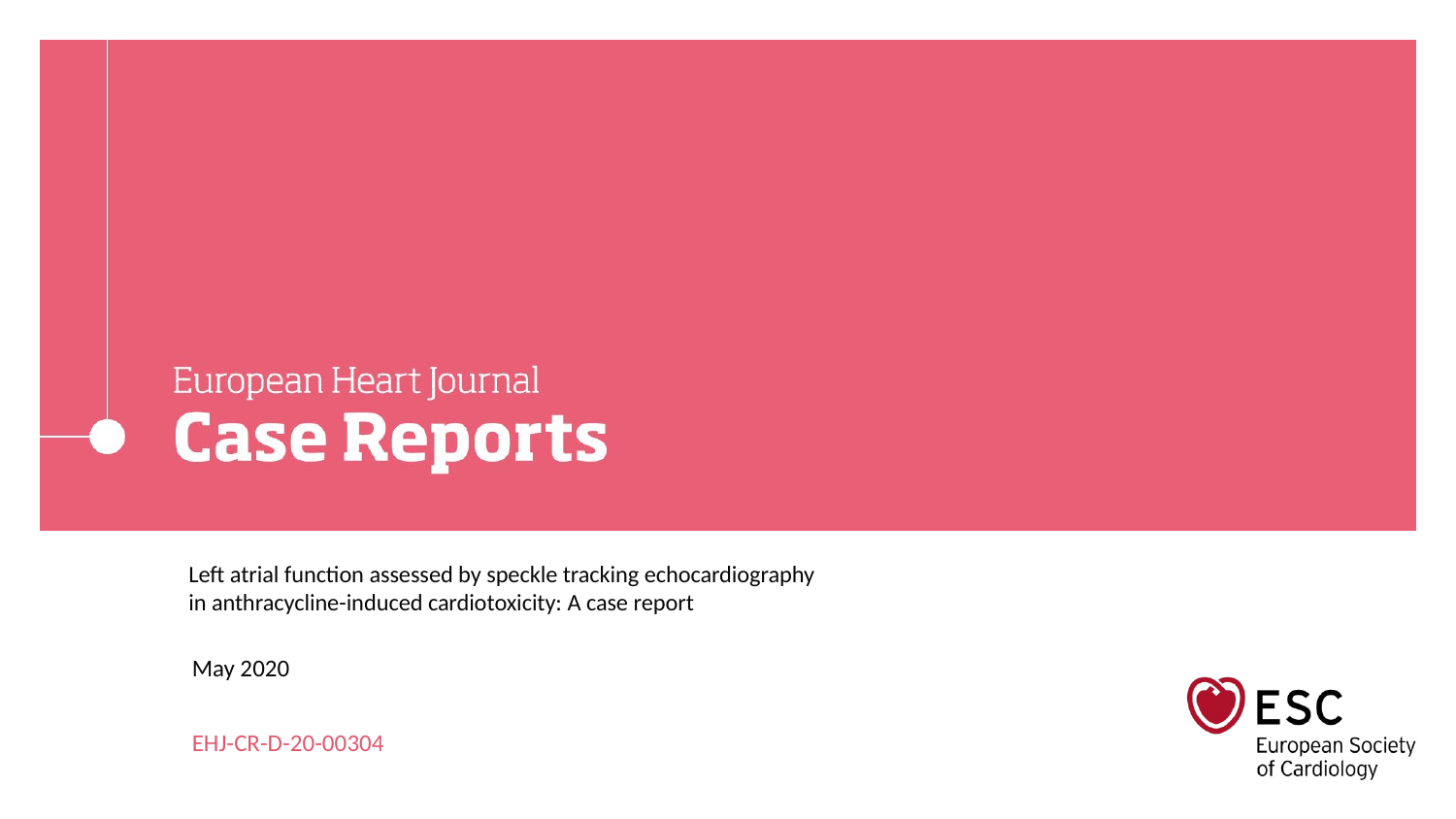

# Left atrial function assessed by speckle tracking echocardiography in anthracycline-induced cardiotoxicity: A case report
May 2020
EHJ-CR-D-20-00304

## Slide 2
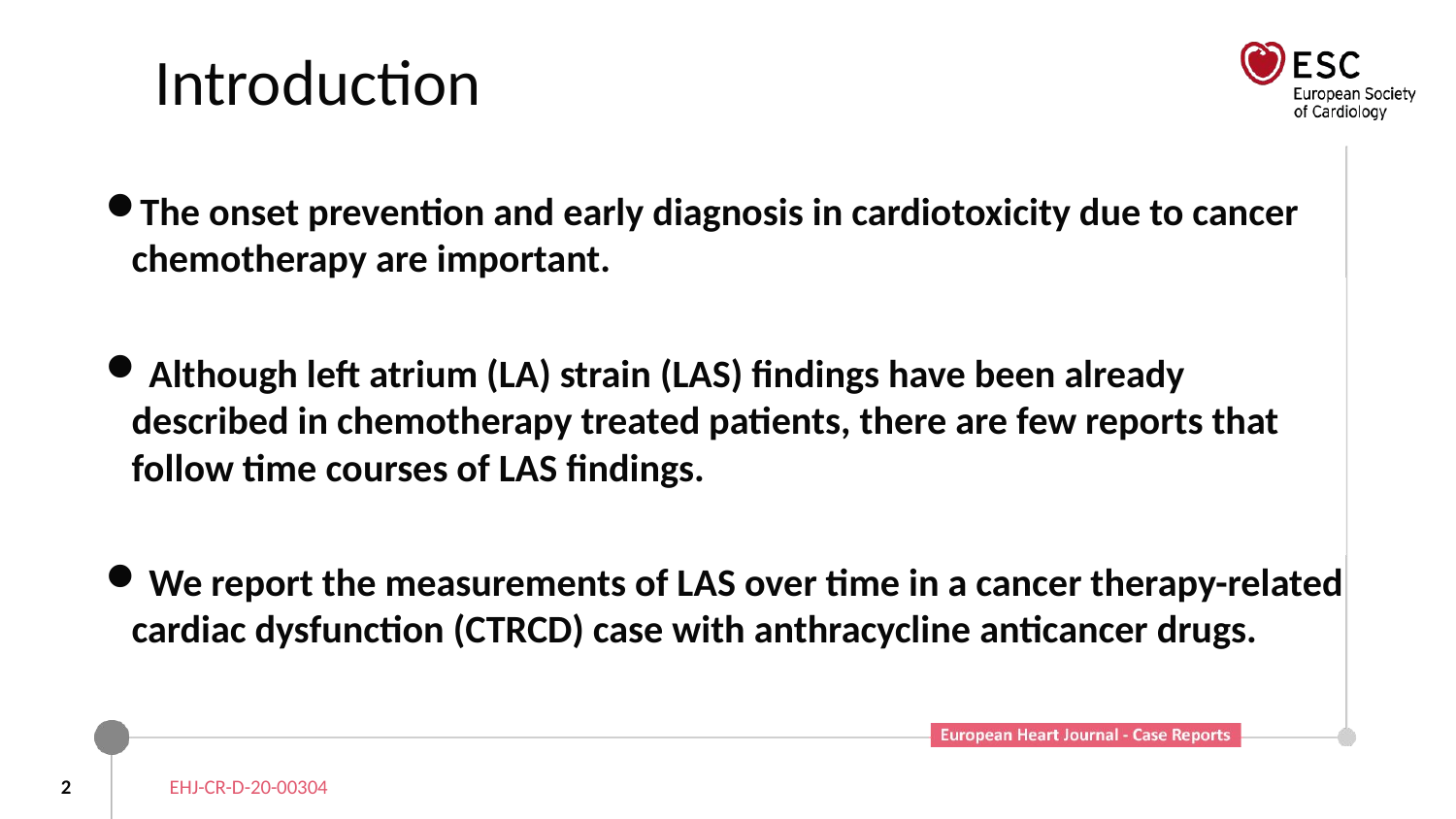

# Introduction
The onset prevention and early diagnosis in cardiotoxicity due to cancer chemotherapy are important.
 Although left atrium (LA) strain (LAS) findings have been already described in chemotherapy treated patients, there are few reports that follow time courses of LAS findings.
 We report the measurements of LAS over time in a cancer therapy-related cardiac dysfunction (CTRCD) case with anthracycline anticancer drugs.
2
EHJ-CR-D-20-00304

## Slide 3
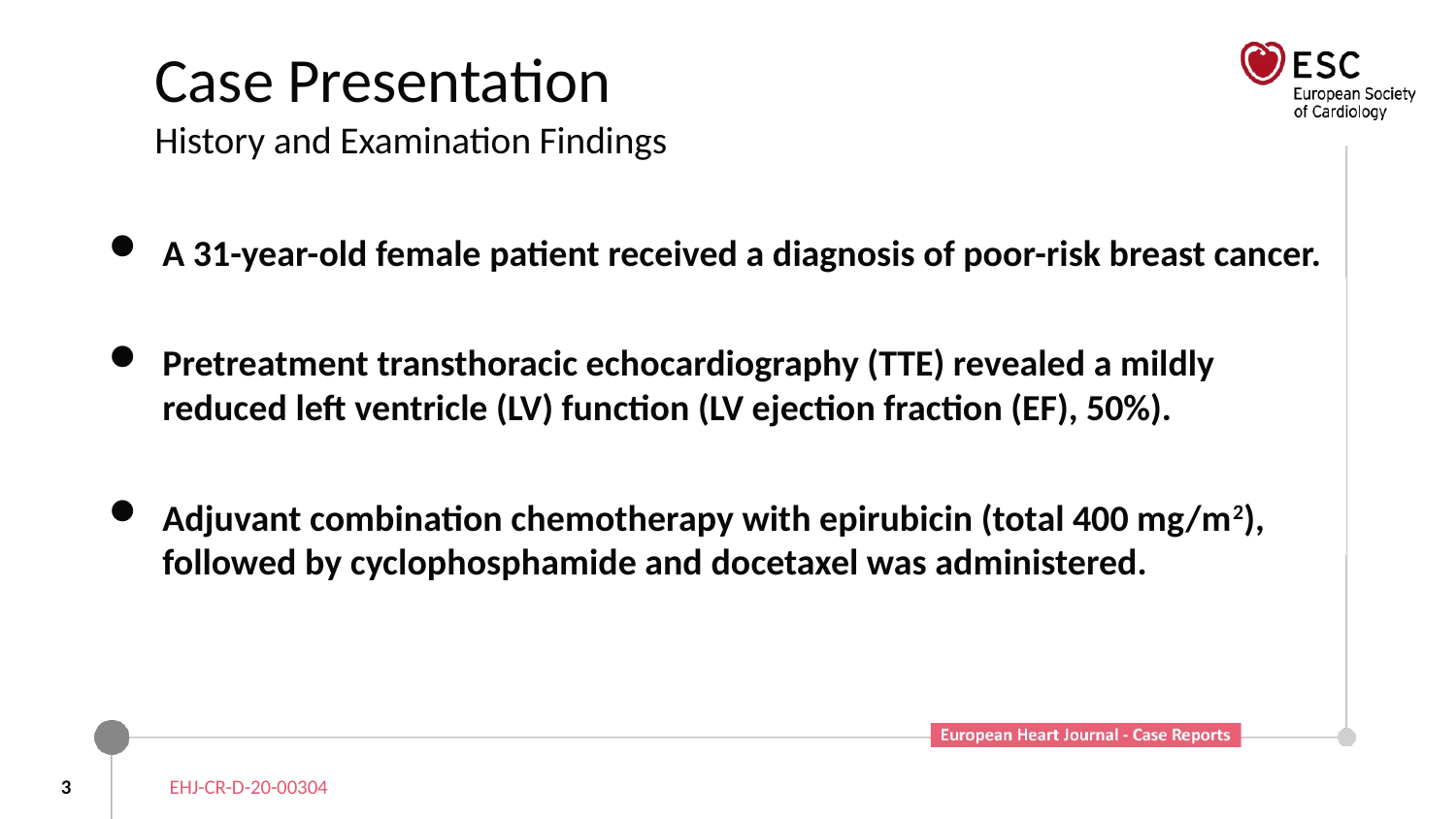

# Case Presentation History and Examination Findings
A 31-year-old female patient received a diagnosis of poor-risk breast cancer.
Pretreatment transthoracic echocardiography (TTE) revealed a mildly reduced left ventricle (LV) function (LV ejection fraction (EF), 50%).
Adjuvant combination chemotherapy with epirubicin (total 400 mg/m2), followed by cyclophosphamide and docetaxel was administered.
3
EHJ-CR-D-20-00304

## Slide 4
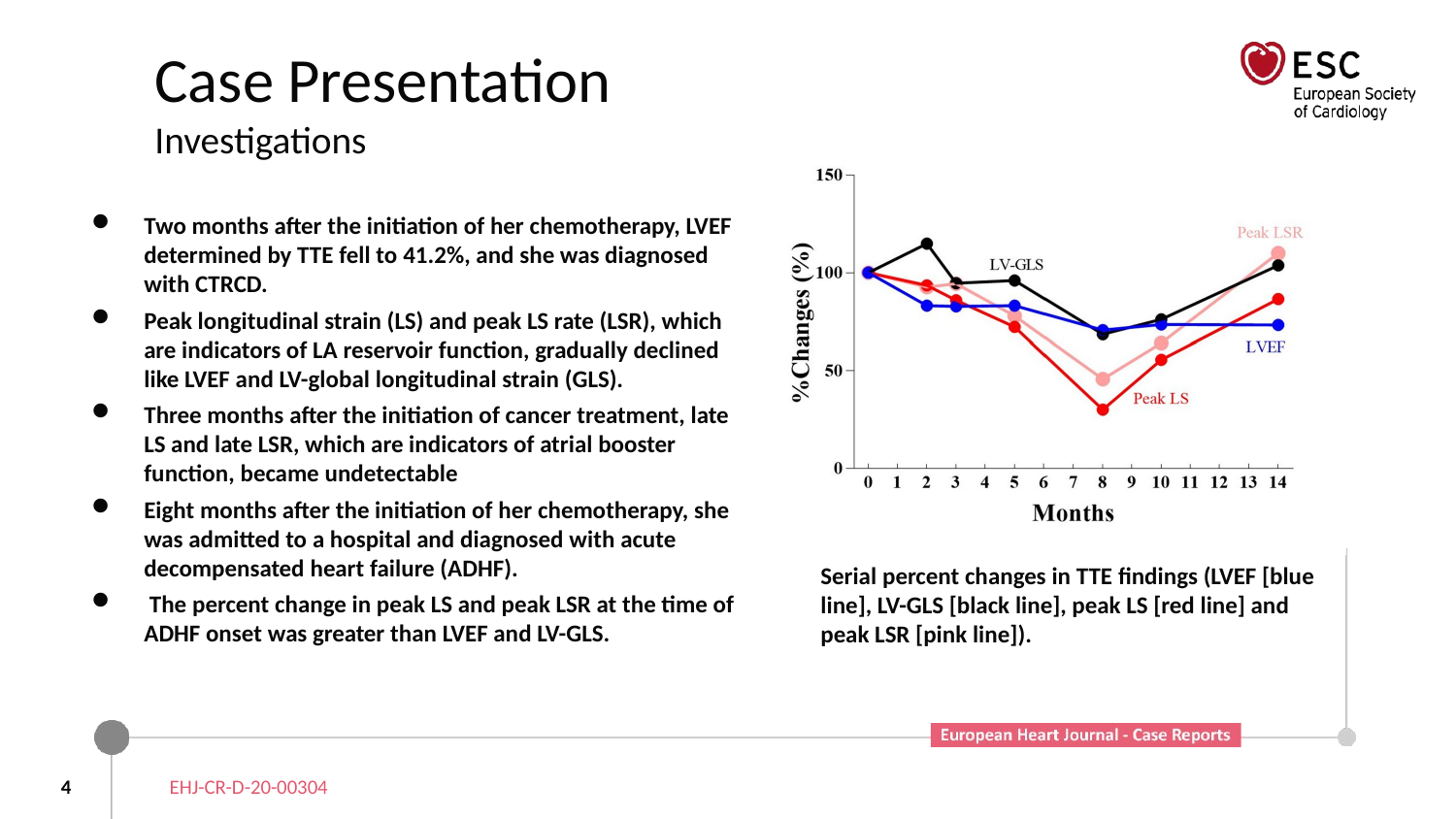

# Case Presentation Investigations
Two months after the initiation of her chemotherapy, LVEF determined by TTE fell to 41.2%, and she was diagnosed with CTRCD.
Peak longitudinal strain (LS) and peak LS rate (LSR), which are indicators of LA reservoir function, gradually declined like LVEF and LV-global longitudinal strain (GLS).
Three months after the initiation of cancer treatment, late LS and late LSR, which are indicators of atrial booster function, became undetectable
Eight months after the initiation of her chemotherapy, she was admitted to a hospital and diagnosed with acute decompensated heart failure (ADHF).
 The percent change in peak LS and peak LSR at the time of ADHF onset was greater than LVEF and LV-GLS.
Serial percent changes in TTE findings (LVEF [blue line], LV-GLS [black line], peak LS [red line] and peak LSR [pink line]).
4
EHJ-CR-D-20-00304

## Slide 5
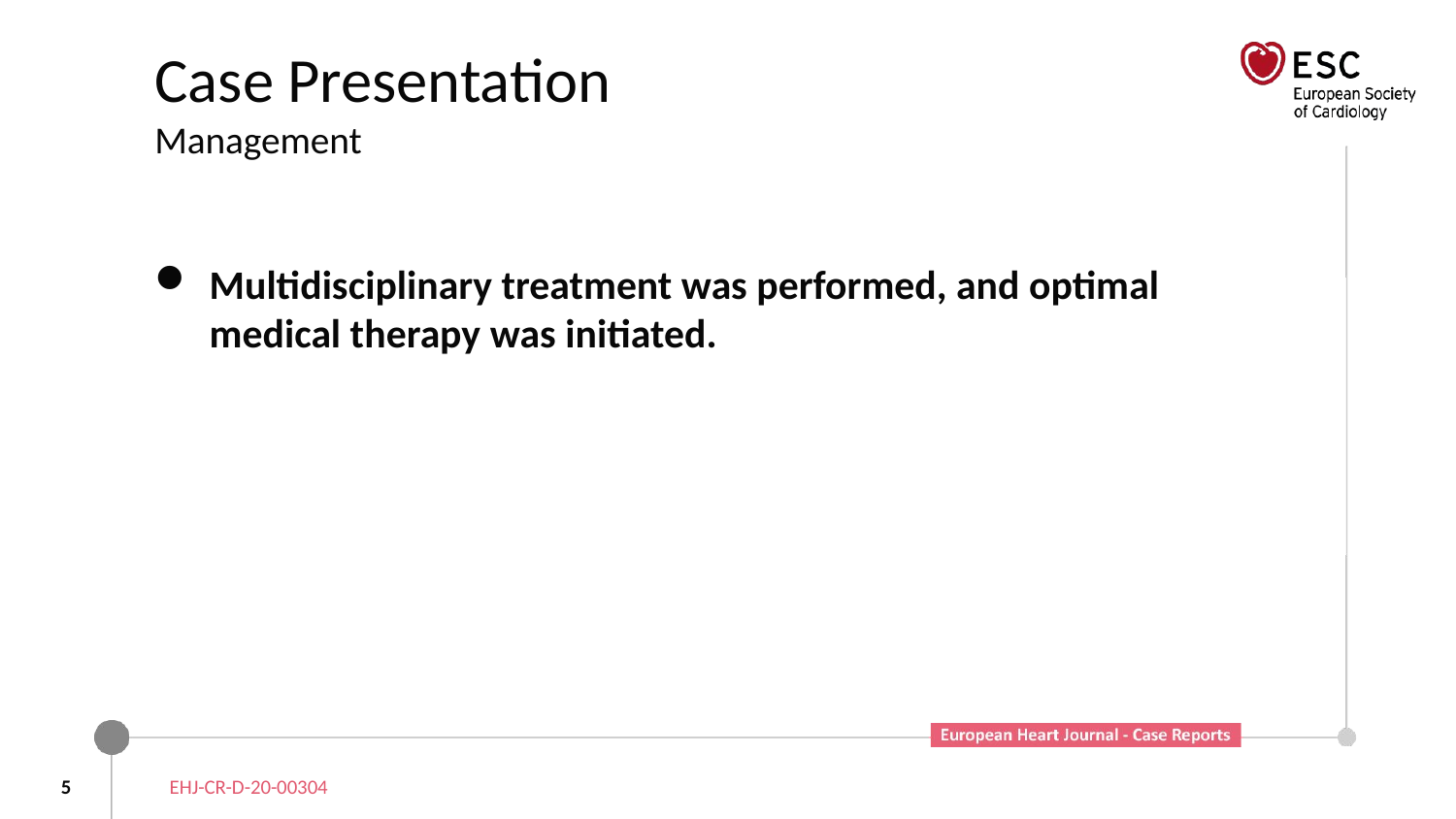

# Case Presentation Management
Multidisciplinary treatment was performed, and optimal medical therapy was initiated.
5
EHJ-CR-D-20-00304

## Slide 6
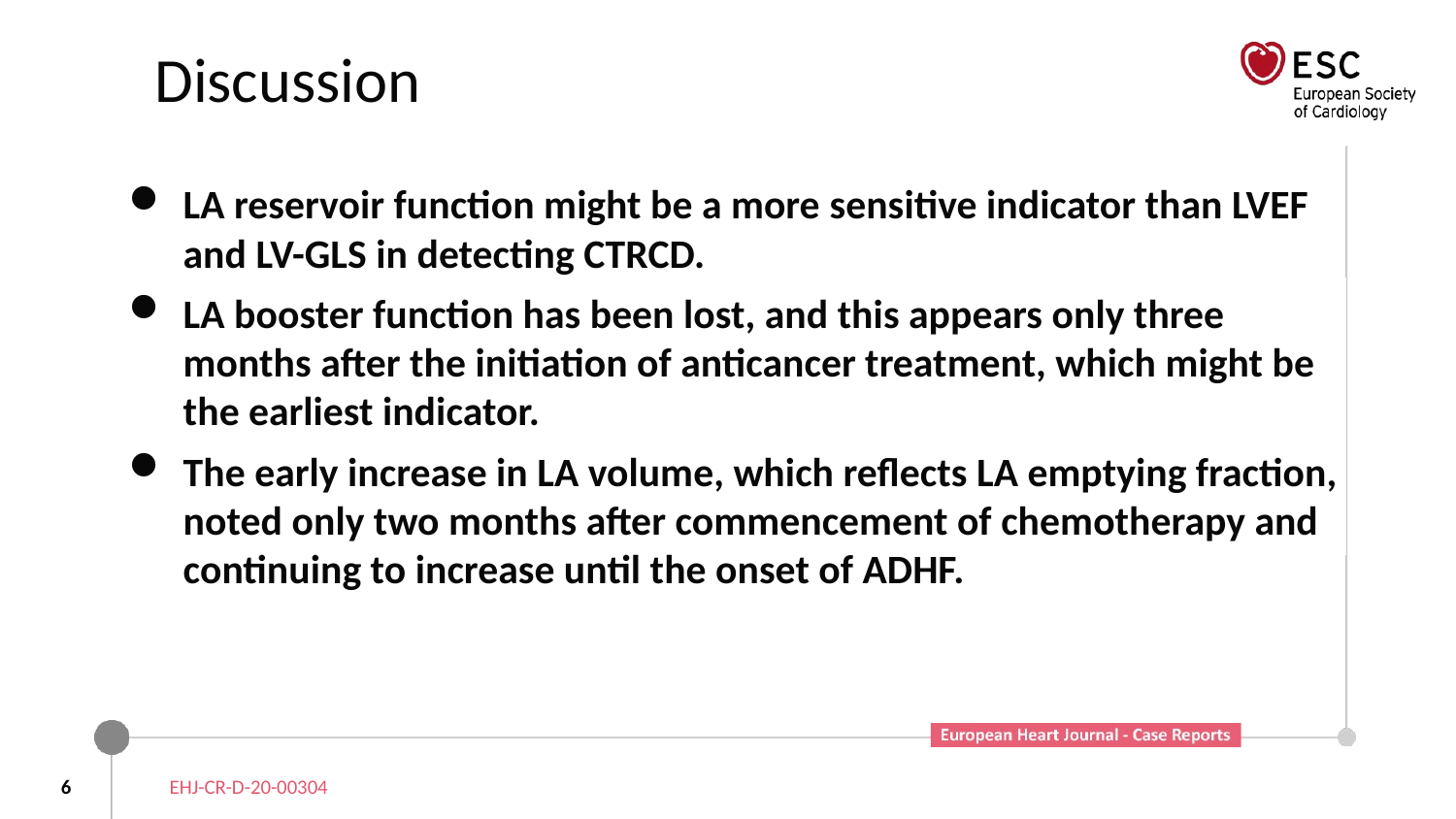

# Discussion
LA reservoir function might be a more sensitive indicator than LVEF and LV-GLS in detecting CTRCD.
LA booster function has been lost, and this appears only three months after the initiation of anticancer treatment, which might be the earliest indicator.
The early increase in LA volume, which reflects LA emptying fraction, noted only two months after commencement of chemotherapy and continuing to increase until the onset of ADHF.
6
EHJ-CR-D-20-00304

## Slide 7
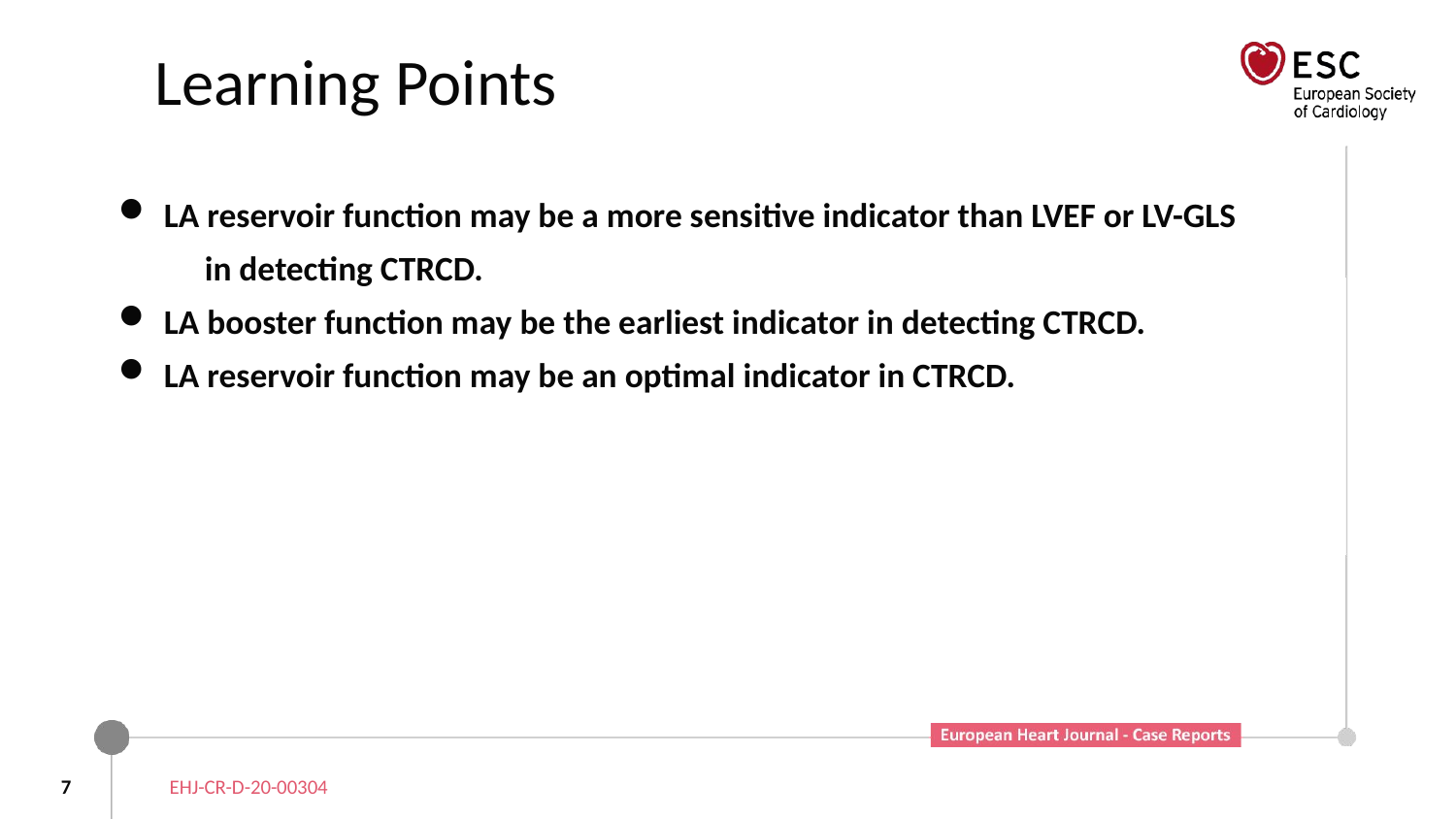

# Learning Points
LA reservoir function may be a more sensitive indicator than LVEF or LV-GLS
　　 in detecting CTRCD.
LA booster function may be the earliest indicator in detecting CTRCD.
LA reservoir function may be an optimal indicator in CTRCD.
7
EHJ-CR-D-20-00304

## Slide 8
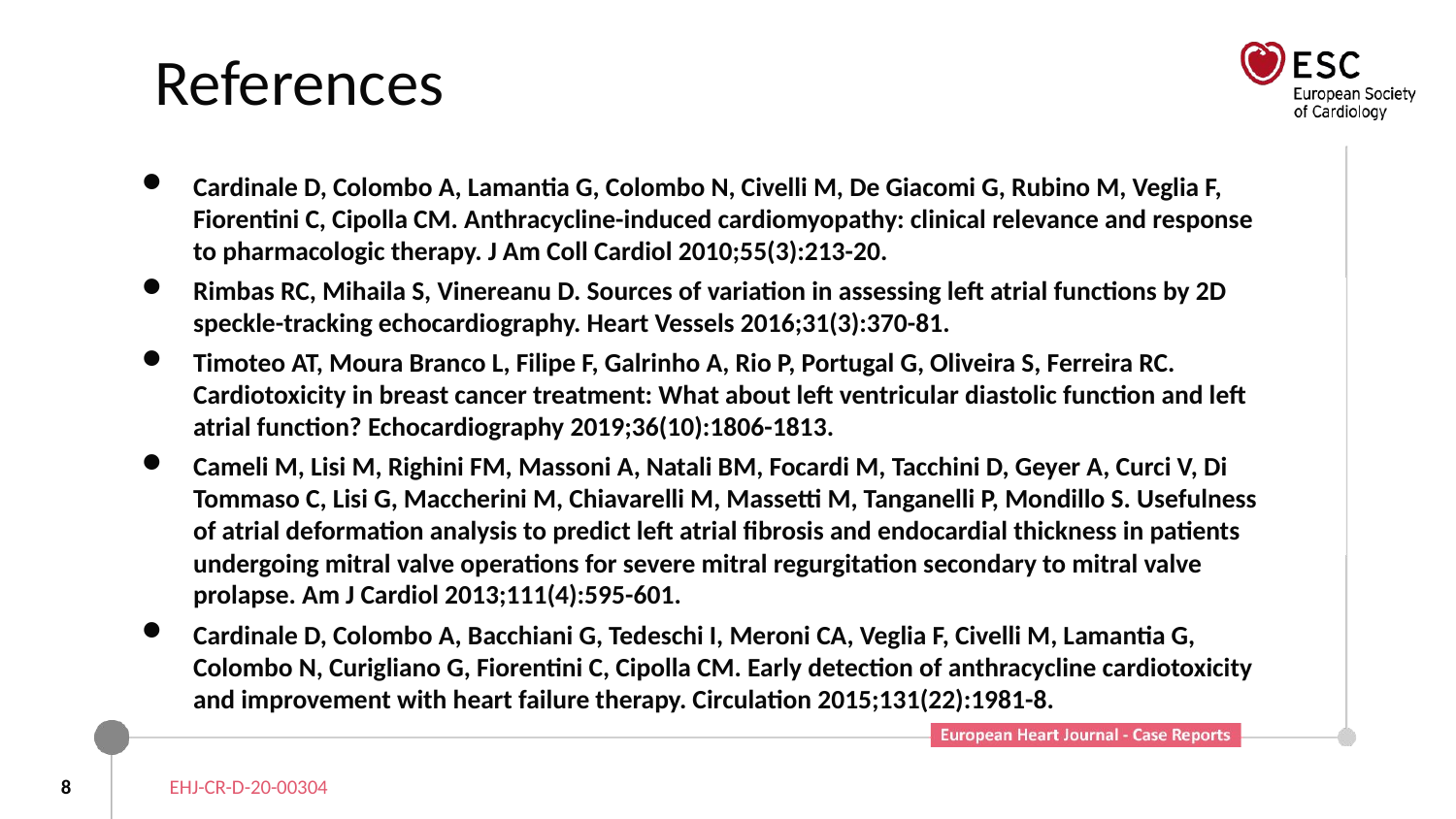

# References
Cardinale D, Colombo A, Lamantia G, Colombo N, Civelli M, De Giacomi G, Rubino M, Veglia F, Fiorentini C, Cipolla CM. Anthracycline-induced cardiomyopathy: clinical relevance and response to pharmacologic therapy. J Am Coll Cardiol 2010;55(3):213-20.
Rimbas RC, Mihaila S, Vinereanu D. Sources of variation in assessing left atrial functions by 2D speckle-tracking echocardiography. Heart Vessels 2016;31(3):370-81.
Timoteo AT, Moura Branco L, Filipe F, Galrinho A, Rio P, Portugal G, Oliveira S, Ferreira RC. Cardiotoxicity in breast cancer treatment: What about left ventricular diastolic function and left atrial function? Echocardiography 2019;36(10):1806-1813.
Cameli M, Lisi M, Righini FM, Massoni A, Natali BM, Focardi M, Tacchini D, Geyer A, Curci V, Di Tommaso C, Lisi G, Maccherini M, Chiavarelli M, Massetti M, Tanganelli P, Mondillo S. Usefulness of atrial deformation analysis to predict left atrial fibrosis and endocardial thickness in patients undergoing mitral valve operations for severe mitral regurgitation secondary to mitral valve prolapse. Am J Cardiol 2013;111(4):595-601.
Cardinale D, Colombo A, Bacchiani G, Tedeschi I, Meroni CA, Veglia F, Civelli M, Lamantia G, Colombo N, Curigliano G, Fiorentini C, Cipolla CM. Early detection of anthracycline cardiotoxicity and improvement with heart failure therapy. Circulation 2015;131(22):1981-8.
8
EHJ-CR-D-20-00304
