# Supplementary figures and images for: Left atrial function assessed by speckle tracking echocardiography in anthracycline-induced cardiotoxicity: a case report
Source: Eur Heart J Case Rep. 2020 Nov 12;4(6):1–5. doi: 10.1093/ehjcr/ytaa355 (PMC7793052; doi:10.1093/ehjcr/ytaa355)

## Slide 1
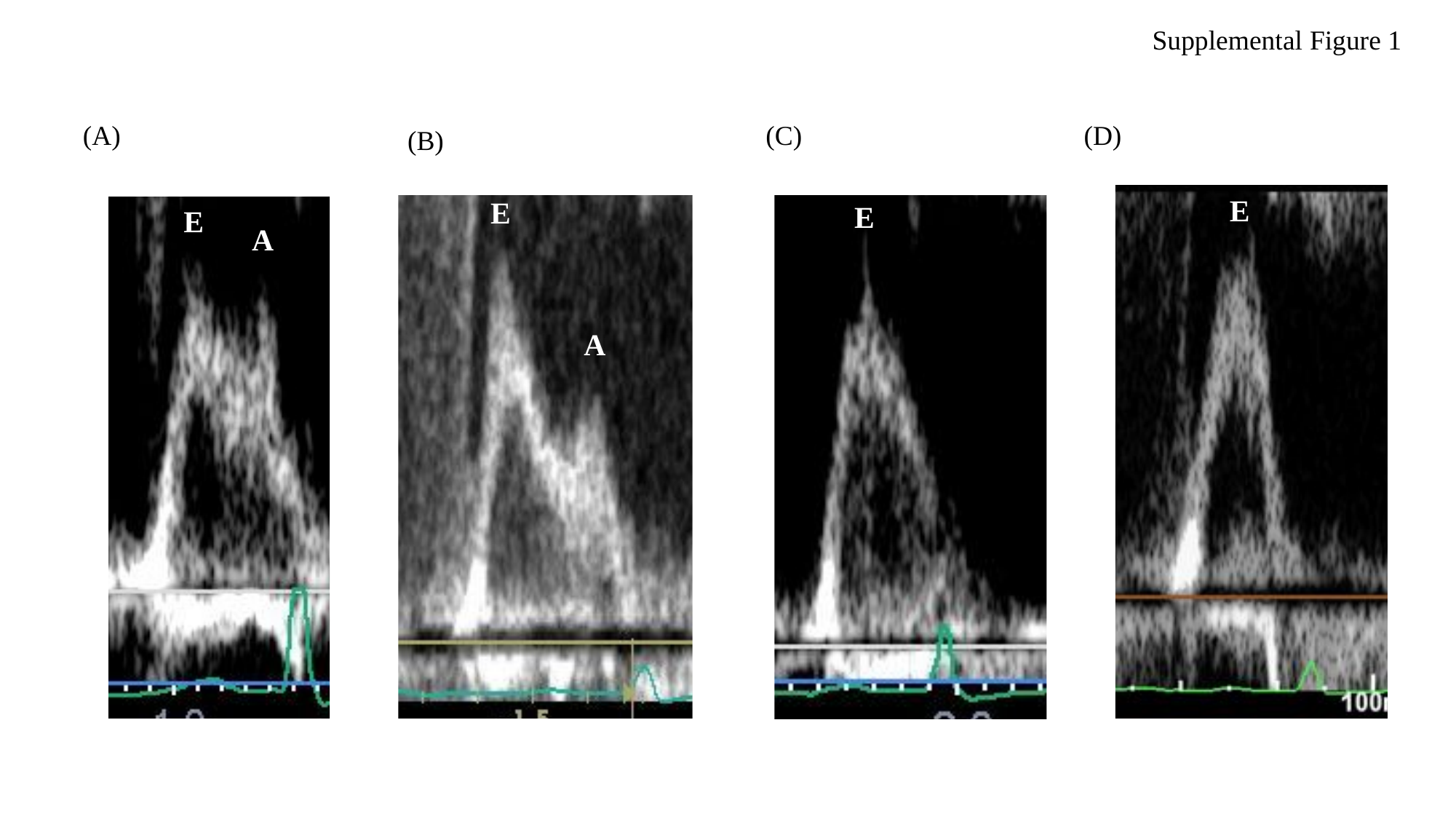

Supplemental Figure 1
(A)
(C)
(D)
(B)
10/30
E
E
E
E
A
A

Supplement: ytaa355_Supplementary_Data [file ytaa355_supplementary_data.zip › ytaa355-suppl_data/Supplememental Figure 1.pptx]
